# The Neural Correlates of Response Inhibition With and Without Conflict in ADHD: An Activation Likelihood Estimation Meta‐Analysis

**DOI:** 10.1111/ejn.70244

**Published:** 2025-09-04

**Authors:** Sarah Daviddi, Valerio Santangelo

**Affiliations:** ^1^ Department of Philosophy, Social Sciences and Education University of Perugia Perugia Italy; ^2^ Functional Neuroimaging Laboratory Fondazione Santa Lucia, IRCCS Rome Italy

**Keywords:** ALE meta‐analysis, attention‐deficit/hyperactivity disorder, caudate, frontal cortex, response inhibition

## Abstract

Attention‐deficit/hyperactivity disorder (ADHD) is a prevalent neurodevelopmental disorder marked—among other features—by impairments in response inhibition, a complex cognitive process assessable through tasks that either involve conflict suppression (C tasks) or do not (no‐C tasks). Previous research has linked impaired response inhibition in ADHD primarily to structural and functional abnormalities in fronto‐striatal and fronto‐parietal networks. However, it remains unclear how these neural circuits differentially support performance on C and no‐C tasks in individuals with ADHD. To address this question, we conducted a meta‐analysis using the activation likelihood estimation (ALE) method. We included the available functional magnetic resonance imaging (fMRI) studies (*N* = 30) examining children and adolescents with ADHD during C and no‐C response inhibition tasks. Across both task types, ADHD participants consistently engaged a fronto‐striatal circuit comprising the inferior frontal gyrus (IFG), anterior cingulate cortex (ACC), and caudate nuclei. When the analysis was restricted on C tasks, activation was primarily observed in the right IFG. In contrast, no‐C tasks elicited activation in the left caudate nucleus, with the additional involvement of the right caudate and ACC during successful response inhibition (i.e., correct performance). These findings reveal a functional dissociation within frontal‐striatal circuits during response inhibition in children and adolescents with ADHD, with the IFG specifically supporting conflict suppression, and the ACC and caudate nuclei contributing more to successful inhibition in tasks without conflict.

## Graphical abstract

The study investigated brain circuits in ADHD during response inhibition. Both conflict (C) and non‐conflict (no‐C) suppression tasks were associated with the fronto‐striatal circuit; C tasks activated the right IFG, whereas no‐C tasks recruited the left caudate nucleus (CN), plus the right CN and ACC during successful response inhibition.

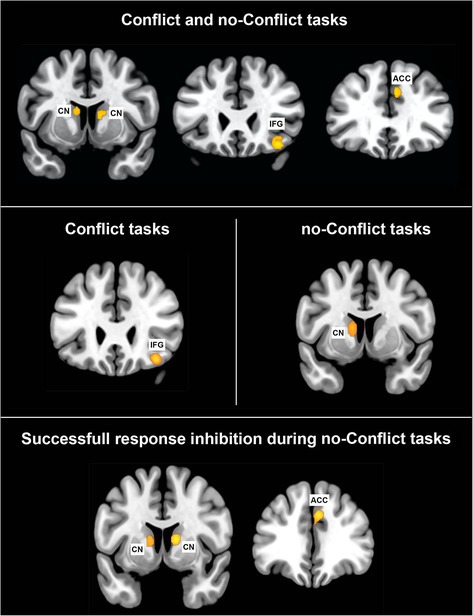

**Abbreviations**
ACC: anterior cingulate cortex
ADHD: attention‐ deficit/hyperactivity disorder
ALE: activation likelihood estimation
C task: conflict tasks
fMRI: functional magnetic resonance imaging
FWE: family‐wise error
FWHM: full‐width half‐maximum
IFG: inferior frontal gyrus
MACM: meta‐analytic connectivity modelling
MNI: Montreal Neurological Institute
No‐C task: no conflict task
PRISMA: Preferred Reporting Items for Systematic Review and Meta‐analysis
ROI: region of interest
SMA: supplementary motor area
TD: typical development

## 1 Introduction

Attention‐deficit/hyperactivity disorder (ADHD) is a neurodevelopmental disorder that affects over 5% of children worldwide and is characterized by persistent inattention, hyperactivity, and impulsivity (Genro et al. 2010; Salari et al. 2023). ADHD symptoms typically arise during childhood and continue into adolescence. Although they can mitigate with age, they often persist into adulthood and can affect academic, occupational, and social functioning (Cortese et al. 2023; McCarthy et al. 2013).

Understanding the underlying neurobiology of ADHD is crucial for developing effective treatment strategies. Recent research has revealed significant differences in the structure and function of the ADHD brain compared to typical development (TD). One of the most consistent findings in ADHD studies is the delayed maturation of frontal regions, which might contribute to account for the mitigation of ADHD symptoms as a function of age (for a review, see Genro et al. 2010). Consistently, several studies showed reduced volumes of frontal, striatal and cerebellar structures in individuals with ADHD (Nakao et al. 2011; for meta‐analyses, see Lukito et al. 2020; Chen et al. 2025). These areas are known to be involved in higher‐order cognitive functions (e.g., planning, organization, and decision‐making), in reward processing and motivation, and in regulating movement and coordination (Salehinejad et al. 2021; Yoshida et al. 2022). The structural abnormalities observed in ADHD brains are reflected at the functional level, mainly in the abnormal activations of fronto‐parietal regions and the fronto‐striatal network. For instance, a recent meta‐analysis (Wang et al. 2025) focused on investigating the neural correlates of executive function and attention in children with ADHD—compared to typically developing peers—found hypoactivation of key areas of the fronto‐parietal network. Similarly, several studies have shown hypoactivation of the fronto‐striatal network, particularly in the prefrontal cortex, caudate nucleus, and putamen (e.g., Booth et al. 2005; Durston et al. 2006; for a meta‐analysis, see Hart et al. 2013). Abnormal activation of the fronto‐striatal network in ADHD has also been found in studies using resting state functional imaging (Chen et al. 2025). This network is primarily involved in the regulation of attention, impulse control, and reward processing, and it involves the dopaminergic system (for reviews, see Tripp and Wickens 2009; Durston et al. 2011). Studies have consistently shown a genetic alteration involving this neurotransmitter in individuals with ADHD (for a review, see Bush 2010). The centrality of the frontal cortex and the dopaminergic system in ADHD is also supported by similarities in symptoms with frontal or Parkinsonian patients (Fan et al. 2020).

One of the core functions sustained by the fronto‐striatal network is response inhibition (Morein‐Zamir and Robbins 2015; Casey et al. 2001), a complex cognitive process involving the ability to resist or suppress an automatic or prepotent response in favour of a more appropriate one (Aron 2007). Response inhibition is a central component of executive functions and plays a crucial role in a wide range of daily activities, including controlling impulsive behaviours (Dillon and Pizzagalli 2007; Friedman and Robbins 2022). According to Barkley (1997), ADHD symptoms may be attributed to a primary inhibition deficit. Consistently, multiple studies demonstrated that individuals with ADHD exhibited poorer performance on tasks measuring response inhibition (e.g., Booth et al. 2005; Durston et al. 2006; for reviews or meta‐analyses, see Weiss and Luciana 2022; Senkowski et al. 2023; Wang et al. 2023). For example, Tamm et al. (2004) showed that adolescents with ADHD committed more errors and omissions than TD participants in a go‐no go task, in which subjects are asked to press or withhold from pressing a button depending on the specific target stimulus presented.

Neuroimaging studies have started to shed light on the neural underpinnings of response inhibition deficits in ADHD, showing a general reduction of activity in the prefrontal cortex (for a review, see Hart et al. 2013), despite some inconsistencies in the literature, likely related to the specific type of task being administered (Hart et al. 2014; Vaidya et al. 2005; Shulz et al. 2005). Response inhibition can be measured in tasks where there is a conflict between stimuli (C tasks); these tasks require the suppression of prepotent and incorrect responses before selecting and acting on a more appropriate response based on the goal of the task (e.g., Stroop task, flanker task). On the other hand, response inhibition can be measured using tasks without conflict (no‐C tasks), in which participants are asked to execute a motor action and stop/withhold it when a specific target stimulus appears, without executing any other action until the next trial (e.g., go‐no go task, stop signal task). Thus, using different tasks, response inhibition can be studied either in terms of suppression of the interference responses (i.e., Stroop‐like and C tasks) or withholding of an ongoing motor response (i.e., go‐no go‐like and no‐C tasks). In line with most studies, we refer here to the former type of response inhibition as “interference inhibition” (e.g., Hart et al. 2013; Zhang et al. 2017), and to the latter type as “motor inhibition” (e.g., Hart et al. 2013; Stevens et al. 2017).

Hart et al. (2013) in their meta‐analysis showed that the right inferior frontal gyrus (IFG), extending to the insula and striatal areas, is involved in both C and no‐C tasks in individuals with ADHD. This provides support for the idea that these areas sustain more general inhibition domains. In contrast, they found that the supplementary motor area (SMA) and the anterior cingulate cortex (ACC) are selectively linked to no‐C tasks. Lei et al. (2015) confirmed the involvement of the SMA, bilateral IFG and right caudate in no‐C tasks in individuals with ADHD. However, they also reported the involvement of posterior regions, specifically, the postcentral gyrus and precuneus. The activation of the fronto‐parietal network in ADHD during response inhibition tasks was also reported by Cortese et al. (2012), peaking in the right parieto‐occipital cortex and the right intermediate frontal sulcus (see also Gow et al. 2012; Sonuga‐Barke 2010, for consistent electroencephalographic findings on the involvement of posterior brain regions). These fronto‐striatal and fronto‐parietal networks were found to have reduced functional connectivity in ADHD patients during response inhibition (Cubillo et al. 2010).

The heterogeneity of these findings necessitates further clarification, particularly regarding the different types of response inhibition. The present meta‐analysis aims to disambiguate this topic by integrating all of the available functional magnetic resonance imaging (fMRI) literature related to the brain activation of children and adolescents with ADHD during different types of response inhibition. We included in the meta‐analysis both studies based on C and no‐C tasks (e.g., Stroop or go‐no go tasks, respectively), but at the same time, we differentiated the contribution of each task. We used an Activation Likelihood Estimation (ALE) meta‐analysis approach, based on the convergence of activation foci across multiple experiments. We conducted four distinct ALE meta‐analyses. The first meta‐analysis included both types of response inhibition tasks, that is, C and no‐C tasks, indexing for general inhibition. The other two meta‐analyses focused on no‐C tasks, distinguishing between brain activation during motor inhibition (e.g., no go vs. go trials), and specific brain activity during correct motor inhibition (e.g., correct no go vs. go trials) to exclude the potential confounds that may occur during unsuccessful inhibition trials. Finally, we conducted another meta‐analysis that included only C tasks, indexing for interference inhibition. It is important to note that due to the lack of sufficient literature investigating this aspect, we could not run a specific meta‐analysis including only successful interference inhibition during C tasks. Then, we used the clusters resulting from the above meta‐analyses as regions of interest (ROIs) to run a connectivity analysis using meta‐analytic connectivity modelling (MACM). This approach enabled us to investigate the specific neural correlates that underlie the distinct types of response inhibition.

## 2 Materials and Methods

### 2.1 Literature Research

**FIGURE 1:**
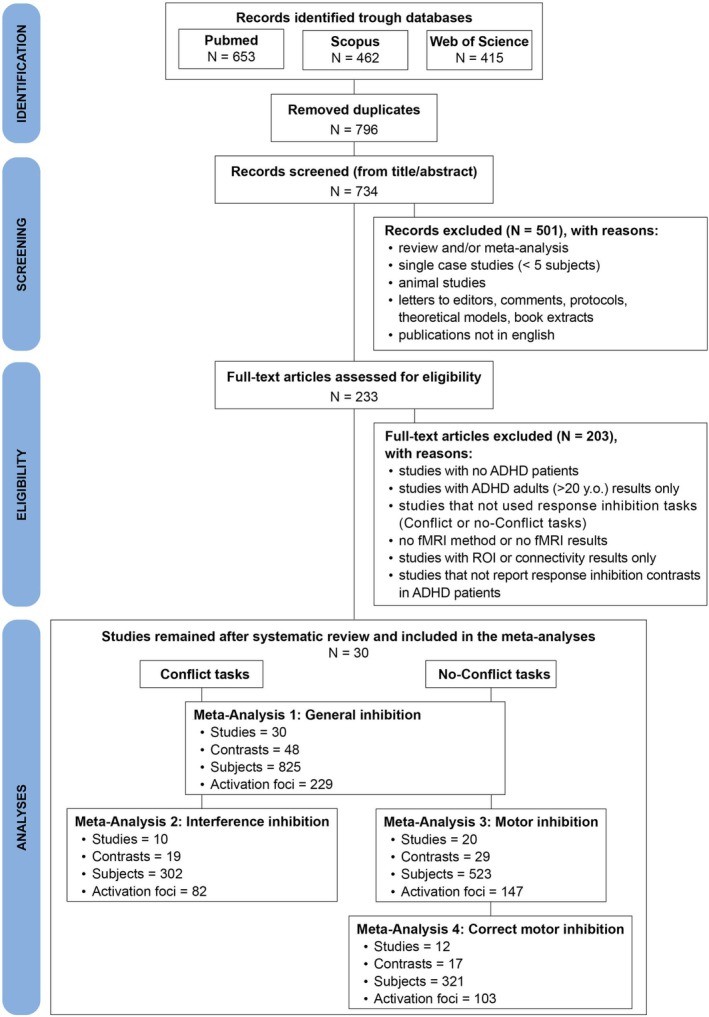
The PRISMA flow chart displays the selection procedure used for the current ALE meta‐analyses, including all the steps of the literature search in detail.

A systematic literature review and article selection process was conducted, following the guidelines outlined in the Preferred Reporting Items for Systematic Review and Meta‐analysis (PRISMA) statement (Page et al. 2021), as shown in Figure 1. This study was not preregistered.

Papers were searched for in three different databases (i.e., Pubmed, Scopus, and Web of Science) using a search string tailored to the scope of our analysis (cf. Harari et al. 2020; Liberati et al. 2009): “fmri OR functional magnetic resonance AND adhd AND task.” The final literature search was conducted on October 4, 2023, and yielded a total of 1530 papers (PubMed: 653; Scopus: 462; Web of Science: 415). After removing duplicate papers, the number of articles was limited to 734. We then proceeded with the selection phase, based on the following inclusion and exclusion criteria: (1) single case studies (with less than 5 subjects), reviews, meta‐analysis, animal studies, book extracts, editorials, letters to editors, protocols, theoretical models, convention abstracts, and publications not in English were excluded; (2) only studies that involved children/adolescents with less than 20 years old and ADHD diagnosis—based on a clinical assessment according to established diagnostic criteria from the DSM (Diagnostic and Statistical Manual of Mental Disorders) or the ICD (International Classification of Diseases)—were included; thus, we excluded studies that only reported on adults with ADHD to reduce heterogeneity and improve interpretability, as ADHD differs across the lifespan in terms of symptoms, compensatory behaviours, and neural correlates (see Leahy 2018; Hoogman et al. 2017); (3) we included articles studying ADHD patients under medication who interrupted the pharmacological consumption at least on the day of the task administration; (4) studies where ADHD patients were administered with tasks required response inhibition (e.g., go‐no go and stop signal task) were included, both with or without conflict (i.e., C or no‐C tasks); (5) only fMRI articles reporting whole‐brain results in Montreal Neurological Institute (MNI) or Talairach space were included; (6) studies reporting only ROIs or connectivity analysis (e.g., dynamic causal modelling) were excluded; (7) only studies reporting contrasts of response inhibition (e.g., stop vs. go for no‐C tasks, or incongruent vs. congruent for C tasks) in ADHD patients were included; (8) only brain activations were included; thus, deactivation results/contrasts were excluded.

Following the PRISMA method and the aforementioned criteria, an initial screening of articles was conducted based on their title and abstract, resulting in the exclusion of 501 papers. Then, we proceeded with the full‐text reading of the 233 remaining papers. Ultimately, a final selection of 30 papers was made (Table 1), ensuring that all inclusion criteria were met. To conduct the meta‐analyses, the coordinates (x, y, z) in 3D stereotactic space and the number of subjects for each selected contrast were extracted from each article. Finally, we reported and examined the main behavioural findings of the selected studies, and whether there were differences in sex and age between the C and no‐C datasets that could bias the results.

**TABLE 1:** The table reports all the studies included in the present ALE meta‐analysis.

Articles	Task	ADHD sample size	TD sample size	Behavioural results	Contrasts	ALE Meta‐analysis
Beauregard and Lévesque 2006	Counting Stroop Task	20 (15 NF group; 5 no NF group)		Acc. interference trials in ADHD ~ 50% RTs not reported	Interference vs. neutral contrast in ADHD (NF group)	GI, II
					Interference vs. neutral contrast in ADHD (NF group)	GI, II
Bédard et al. 2010	Go‐No go	33		Acc. no go trials in ADHD > 70%	Response inhibition in ADHD	GI, MI, CMI
Bédard et al. 2015	Go‐No go	25		Acc. no go trials in ADHD ~ 90%	Correct no go vs. correct go in ADHD	GI, MI, CMI
Bhaijiwala et al. 2014	Stop Signal Task	12	12	Acc. stop trials in ADHD ~ 50% Acc. stop trials: ADHD ≅ TD RTs go trials: ADHD > TD	Reacting inhibition in ADHD	GI, MI, CMI
					Reacting inhibition in ADHD > TD	GI, MI, CMI
Booth et al. 2005	Go‐No go	12	12	Acc. no go trials in ADHD ~ 80% Acc. no go trials: ADHD < TD RTs go trials: ADHD ≅ TD	No go minus go in ADHD	GI, MI
Cerullo et al. 2009	Continuous Performance Task	10	13	Acc. stop trials in ADHD ~ 80% Acc. stop trial: ADHD ≅ TD RTs go trial: ADHD ≅ TD	Successful response inhibition in ADHD vs. TD	GI, MI, CMI
Durston et al. 2006	Go‐No go	11	11	Overall acc. in ADHD > 80% Overall acc.: ADHD < TD Overall acc. RTs: ADHD ≅ TD	No go > go in ADHD	GI, MI
Ercan et al. 2016	Go‐No go	100 (50 ADHD‐RI; 50 ADHD‐I)	100	Overall acc. in ADHD > 80% Overall acc.: ADHD < TD Overall acc. RTs: ADHD > TD	No go trials in ADHD ‐ RI > TD	GI, MI
L.‐Y. Fan et al. 2014	Counting Stroop Task	25	23	Acc. incongruent trials in ADHD > 80% Acc. incongruent trials: ADHD ≅ TD RTs incongruent trials: ADHD > TD	Incongruent vs. congruent condition in ADHD	GI, MI
L.‐Y. Fan et al. 2017	Counting Stroop Task	27	54 (27 unaffected siblings; 27 TD)	Acc. incongruent trials in ADHD > 90% Acc. incongruent trials: ADHD ≅ TD RTs incongruent trials: ADHD ≅ TD	Incongruent vs. congruent condition in ADHD > TD	GI, II
					Incongruent vs. congruent condition in ADHD > unaffected siblings	GI, II
Hart et al. 2014	Stop task	30	30	Acc. stop trials in ADHD ~ 50% Acc. stop trials: ADHD ≅ TD RTs go trials: ADHD ≅ TD	Successful stop > go trials in ADHD > TD	GI, MI, CMI
Iannacone et al. 2015	Modified Flanker Task	18	18	Overall acc.: ADHD < TD Overall RTs: ADHD ≅ TD	Inhibition in ADHD > TD	GI, MI, CMI
					Inhibition in ADHD	GI, MI, CMI
Ivanov et al. 2012	Anticipation Conflict Reward Task	20		Acc. incongruent trials < acc. Congruent trials in ADHD RTs incongruent trials > RTs congruent trials in ADHD	Incongruent flanker minus congruent flanker in ADHD (regardless the reward)	GI, II
Ma et al. 2012	Go‐No go	15	15	Overall acc. in ADHD > 90% Overall acc.: ADHD ≅ TD RTs go trials: ADHD ≅ TD	No go minus go in ADHD vs. TD	GI, MI
Massat et al. 2018	Stop Signal Task	18	19	Acc. stop trials in ADHD ~ 46% Acc. stop trials: ADHD ≅ TD RTs go trials: ADHD ≅ TD	Response inhibition in ADHD > TD	GI, MI, CMI
					Response inhibition in ADHD	GI, MI, CMI
Mercadillo et al. 2012	Counting Stroop Task	12		Acc. interference trials < acc. Neutral trials in ADHD RTs not reported	Execution of the task in ADHD	GI, II
Passarotti et al. 2010	Response Inhibition Task	11	15	Acc. stop trials in ADHD > 75% Acc. stop trials: ADHD < TD RTs go trials: ADHD ≅ TD	Stop vs. go in ADHD	GI, MI
					Stop vs. go in ADHD > TD	GI, MI
Posner et al. 2011	Stroop Task	15	15	Acc. number words in ADHD ~ 75% Acc. number words: ADHD < TD RTs number words: ADHD > TD	Number words > neutral words in unmedicated ADHD	GI, II
Saenz et al. 2020	Stop Signal Task	18	14	Acc. stop trials in ADHD ~ 47% Acc. stop trials: ADHD ≅ TD RTs go trials: ADHD > TD	Inhibition in ADHD	GI, MI, CMI
Shang et al. 2022	Counting Stroop Task	49	28	Acc. incongruent trials in ADHD > 90% Acc. incongruent trials: ADHD ≅ TD RTs incongruent trials: ADHD > TD	Incongruent vs. congruent in ADHD pre‐treatment	GI, MI
					Incongruent vs. congruent in ADHD pre‐treatment > TD	GI, MI
Shulz et al. 2012	Go‐No go	36		Acc. no go trials in ADHD > 70%	Correct no go responses in ADHD	GI, MI, CMI
Shulz et al. 2005	Stimulus and Response Conflict Task	8	8	Acc. all conflict trials in ADHD > 85% Acc. all conflict trials: ADHD ≅ TD RTs all conflict trials: ADHD ≅ TD	Stimulus conflict minus control trials in ADHD	GI, II
					Response conflict minus control trials in ADHD	GI, II
					Combined conflict minus control trials in ADHD	GI, II
					Stimulus conflict minus control trials in ADHD vs. TD	GI, II
					Combined conflict minus control trials in ADHD > TD	GI, II
Shulz et al. 2004	Go‐No go	10	9	Acc. no go trials in ADHD ~ 70% Acc. no go trials: ADHD < TD RTs go trials: ADHD ≅ TD	Correct no go vs. correct go in ADHD > TD	GI, MI, CMI
					Correct no go vs. correct go in ADHD	GI, MI, CMI
Siniatchkin et al. 2012	Go‐No go	12	12	Acc. no go trials: ADHD ≅ TD RTs go trials: ADHD > TD	Activation during no go condition in ADHD (T1)	GI, MI
Solanto et al. 2009	Go‐No go	20		Acc. no go trials in ADHD ~ 60%	Inhibitory control in ADHD	GI, MI, CMI
Stevens et al. 2017	Go‐No go	62 (22 ADHD‐EF; 16 ADHD‐EF/REW; 24 ADHD‐NONE)	63	Acc. no go trials: ADHD < TD RTs go trials: ADHD ≅ TD	ADHD‐NONE > TD (motor inhibition)	GI, MI, CMI
Suskauer et al. 2008	Go‐No go	25	25	Acc. no go trials in ADHD ~ 75% Acc. no go trials: ADHD ≅ TD RTs go trials: ADHD ≅ TD	ADHD activation during no go trials	GI, MI
					ADHD > TD during no go trials	GI, MI
Tamm et al. 2004	Go‐No go	10	12	Acc. no go trials: ADHD < TD RTs go trials: ADHD ≅ TD	No go vs. go in ADHD	GI, MI
					No go vs. go in ADHD vs. TD	GI, MI
Vaidya et al. 2005	Modified Flanker Task	10	10	Acc. incongruent trials in ADHD > 85% Acc. incongruent trials: ADHD < TD RTs incongruent trials: ADHD ≅ TD	Incongruent vs. neutral task in ADHD	GI, II
					Incongruent vs. neutral task during successful suppression in ADHD	GI, II
					No go vs. neutral trials in ADHD	GI, MI
					No go vs. neutral trials during successful suppression in ADHD	GI, MI, CMI
Zamorano et al. 2017	Multi Source Interference Task	17	17	Overall acc.: ADHD < TD Overall acc. RTs: ADHD ≅ TD	Incongruent vs. congruent trials in ADHD > TD	GI, II

*Note:* From the left, for each study we reported the task, ADHD and TD sample size, main behavioural results (mean accuracy and RTs), and contrasts used in the meta‐analysis. In the last column, we specified in which analysis (GI = General Inhibition; II = Interference Inhibition; MI = Motor Inhibition; CMI = Correct Motor Inhibition) we used each contrast.

Abbreviations: Acc.: accuracy; ADHD: attention deficit‐hyperactivity disorder; ADHD‐EF/REW: ADHD impaired on both Executive Function and reward tests; ADHD‐EF: ADHD impaired on Executive Function test only; ADHD‐I: ADHD predominantly inattentive; ADHD‐NONE: ADHD largely intact on dual‐pathway tests; ADHD‐RI: ADHD‐restrictive inattention; NF: neurofeedback; RTs: reaction times; TD: typical development.

### 2.2 ALE Meta‐Analysis

Consistent with existing literature (Daviddi et al. 2023; Eickhoff et al. 2016), the term “experiment” was used to denote the individual contrast within the ALE meta‐analysis, while the term “study” was used to refer to distinct papers or articles. The ALE meta‐analysis was conducted to assess the convergence of activation foci above chance across experiments, thereby rejecting the null hypothesis of a random spatial distribution of these foci in the brain (Eickhoff et al. 2012). This meta‐analytical approach models foci from each experiment using a Gaussian probability distribution, capturing the spatial uncertainty associated with each single focus (Eickhoff et al. 2009, 2012). The full‐width half‐maximum (FWHM) value was empirically determined based on the sample subject size of each experiment (Eickhoff et al. 2009). A voxel‐wise modelled activation map was generated from the probability distribution of activation foci in each experiment (Eickhoff et al. 2012). The distinct modelled activation maps were merged to compute ALE scores. The resulting ALE map was then compared against a null distribution (Eickhoff et al. 2012).

GingerALE 3.0.2 (https://brainmap.org/ale/) was utilized to transform all Talairach foci into MNI coordinates and to conduct the analyses. No custom‐made code was used for the analyses in this study. Four distinct meta‐analyses were carried out: (1) the first ALE examined general response inhibition in individuals with ADHD, irrespective of response correctness or type of inhibition (i.e., general inhibition analysis; including 48 experiments, 825 subjects, and 229 foci); (2) the second ALE examined interference inhibition in individuals with ADHD during C tasks, irrespective of response correctness (i.e., interference inhibition analysis; including 19 experiments, 302 subjects, and 82 foci); (3) the third ALE examined response inhibition in individuals with ADHD during no‐C tasks, irrespective of response correctness (i.e., motor inhibition analysis; including 29 experiments, 523 subjects, and 147 foci); and finally, (4) the fourth ALE examined correct response inhibition in individuals with ADHD during no‐C tasks, thus including correct trials only (i.e., correct motor inhibition analysis; including: 17 experiments, 321 subjects, and 103 foci). As per established practices in the field, we have taken care to ensure the reliability of the statistical results for the ALE meta‐analyses by including a minimum of 17 experiments (cf. Eickhoff et al. 2016). The statistical parameters were set to: family‐wise error (FWE) cluster‐level threshold of *p* < 0.05, with clusters estimated at an uncorrected threshold of *p* < 0.001, with 1000 permutations and no volume restrictions. Finally, we also performed a direct subtraction analysis comparing C (i.e., interference inhibition) and no‐C conditions (i.e., motor inhibition), setting a *p*‐value < 0.001, with 10,000 permutations and no volume restrictions. To ensure anatomical specificity, we applied in these and the following analyses (Section 2.3) a standard grey matter mask to exclude activation in white matter regions from the ALE results, using the SPM12 software package (http://www.fil.ion.ucl.ac.uk/spm) implemented in Matlab R2018b.

### 2.3 MACM

Meta‐analytic connectivity modelling (MACM) enables the investigation of the functional connectivity of specific brain areas by computing the co‐activation patterns across participants of a given seed region (Robinson et al. 2010; Langner et al. 2014). In this study, MACM was used to investigate the functional connectivity of activations resulting from the above ALE meta‐analyses (cf., par. 2.2). Each activation was used as an input ROI in the BrainMap database using Sleuth 3.0.4 (http://www.brainmap.org/sleuth/readme.html), with the following search criteria: “Context: Normal mapping”, “Activation: Activation only” and “Behavioral domain: Action: Inhibition.” As regards the general inhibition analysis, a total of 12 experiments from 9 studies were obtained using the right IFG as ROI, while results from the right ACC ROI consisted of 8 experiments from 6 papers. Concerning the correct motor inhibition analysis, a total of 11 experiments from 9 studies were obtained using the left caudate as ROI, while results from the right ACC ROI consisted of 8 experiments from 8 papers. Searches that produced less than 8 experiments (as those derived from the activations of some of the general inhibition and correct motor inhibition clusters or from the interference inhibition and motor inhibition analysis) were excluded from this MACM analysis due to statistical reasons (Eickhoff et al. 2016). The coordinates of activation of these experiments were downloaded and used to perform the ALE meta‐analysis. Due to the limited number of experiments resulting from the above procedure (less than 17 experiments), a voxel‐level threshold was set up at a p‐FWE‐corrected < 0.05, with 5000 permutations, and a minimum cluster volume of 200 mm^3^ (cf. Eickhoff et al. 2016).

## 3 Results

### 3.1 Main Behavioural Findings

First, potential demographic differences between the participants in the C and no‐C studies were examined. Regarding sex, both the C and no‐C datasets showed comparable male predominance. Most no‐C studies reported a higher proportion of male participants, except for one study that reported 44% males (e.g., Massat et al. 2018) and one study that did not specify the sex distribution (e.g., Bhaijiwala et al. 2014). Similarly, all C studies reported a male majority. A chi‐square test confirmed that the proportion of male participants did not differ significantly between the two datasets (*χ* ^2^ = 0.568, *p* > 0.05). As for age, no significant difference in mean age was found between the two datasets (t = 1.432, *p* > 0.05). Overall, these findings indicate that the C and no‐C samples are comparable in terms of age and sex.

Next, we examined potential behavioural patterns across the selected studies, as a basis for the related neural findings, which are the main focus of this study. Table 1 summarizes the main behavioural findings of each article included in the ALE meta‐analysis. Behavioural results were reported heterogeneously across the included studies. Collectively, in the no‐C dataset, children with ADHD generally showed relatively high accuracy in go‐no go tasks, around or above 70% (except for one study: Solanto et al. 2009), while accuracy in stop signal tasks tended to remain around 50%. Reaction times (RTs) between go and no‐go trials could not be compared because no go trials require no response, as do stop signal tasks. In the C dataset (including congruent/incongruent paradigms), children with ADHD generally showed accuracy of about or above 65% (with one exception: Beauregard and Lévesque 2006) for incongruent trials. When comparing incongruent vs. congruent trials, children with ADHD performed worse in the former and exhibited slower RTs in incongruent compared to congruent trials. Finally, in the studies that directly compared ADHD and TD children, the ADHD group generally performed worse or, at most, performed similarly to the TD group in terms of accuracy and RTs (see Table 1, “Behavioural results” column).

### 3.2 Single Contrast ALE Meta‐Analysis

**FIGURE 2:**
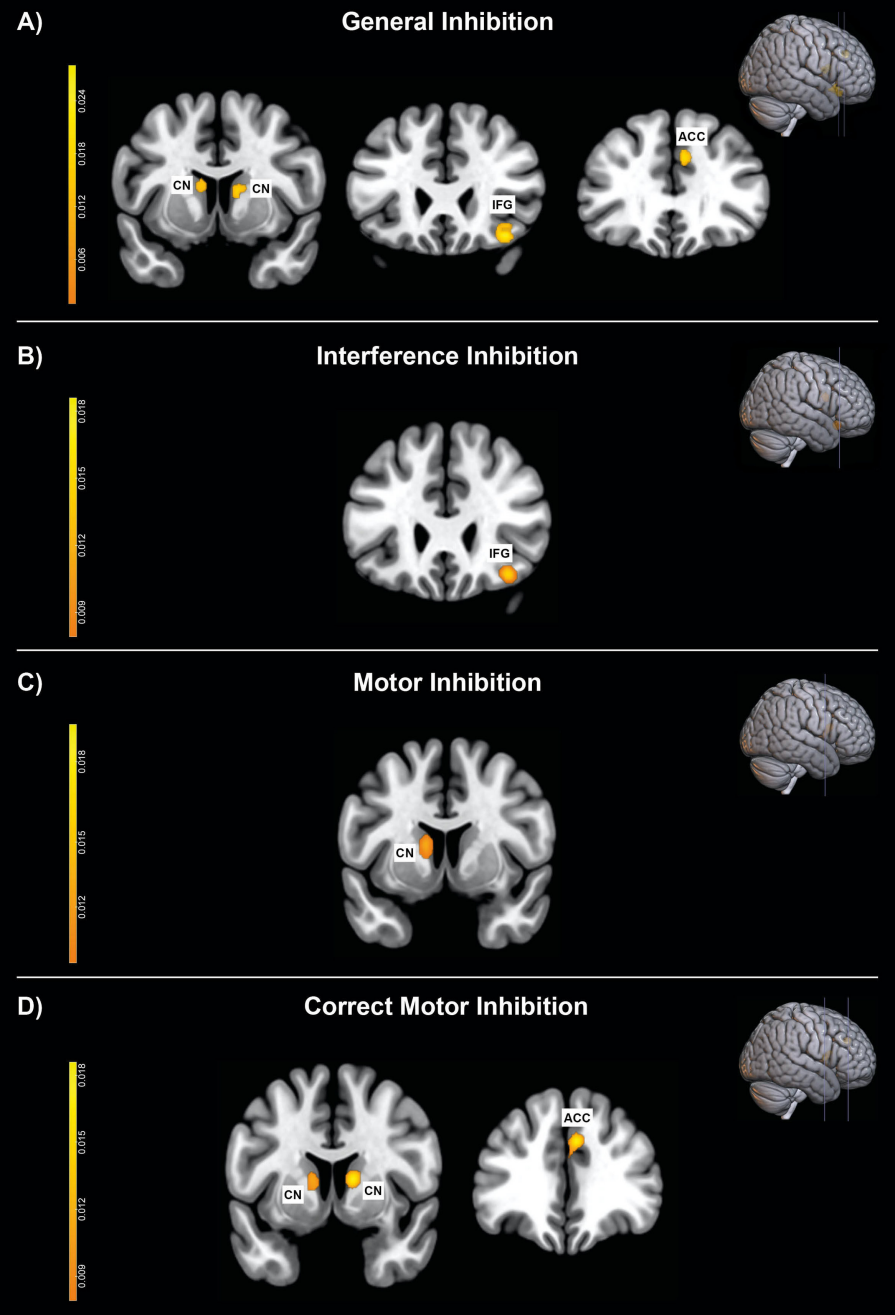
The image shows the results of all the ALE meta‐analyses conducted. (A) General inhibition, showing four clusters of activation in the left and right caudate nuclei (CN), right anterior cingulate cortex (ACC), and right inferior frontal gyrus (IFG). (B) Interference inhibition, showing a cluster of activation in the right IFG. (C) Motor inhibition, showing a cluster of activation located in the right CN. (D) Correct motor inhibition, showing three clusters of activation in the bilateral CN and right ACC.

The results of the ALE meta‐analysis conducted on general inhibition contrasts showed activation in several brain regions, including the right IFG (pars orbitalis; cluster 1), the right ACC (cluster 3), the left and the right caudate nucleus (respectively, clusters 2 and 4) (Table 2A and Figure 2A). The second ALE meta‐analysis conducted on interference inhibition contrasts revealed activation in the right IFG (pars orbitalis; cluster 1 in Table 2B; Figure 2B). The results of the third ALE meta‐analysis conducted on motor inhibition contrasts showed one single cluster of activation in the left caudate (Table 2C and Figure 2C). Finally, the fourth ALE meta‐analysis conducted on correct motor inhibition contrasts revealed three distinct clusters of activation (Table 2D and Figure 2D): one located in the right ACC (cluster 2), and the other two in the left and right caudate nucleus (clusters 1 and 3, respectively). Lastly, the direct subtraction analysis comparing C and no‐C conditions yielded no significant results.

**TABLE 2:** ALE meta‐analysis results. From the left, the table reports number, region, hemisphere, volume in mm^3^, ALE score, and coordinates for each cluster.

Cluster #	Region	Hemisphere	Volume (mm^3^)	ALE score	x	y	z
(A) General inhibition							
1	IFG (pars orbitalis)	R	1920	0.028	36	24	−16
1		R		0.016	42	14	−14
2	Caudate	L	1444	0.019	−8	12	16
		R		0.015	4	8	20
3	ACC	R	952	0.024	6	34	32
4	Caudate	R	768	0.021	12	6	10
(B) Interference inhibition							
1	IFG (pars orbitalis)	R	824	0.018	36	22	−16
(C) Motor inhibition							
1	Caudate	L	1128	0.019	−8	12	16
(D) Correct motor inhibition							
1	Caudate	L	1256	0.015	−8	12	16
1		L		0.011	−18	6	2
2	ACC	R	928	0.019	6	34	32
2		R		0.010	2	36	22
3	Caudate	R	792	0.020	12	6	10

Abbreviations: ACC: anterior cingulate cortex; IFG: inferior frontal gyrus.

### 3.3 MACM

**FIGURE 3:**
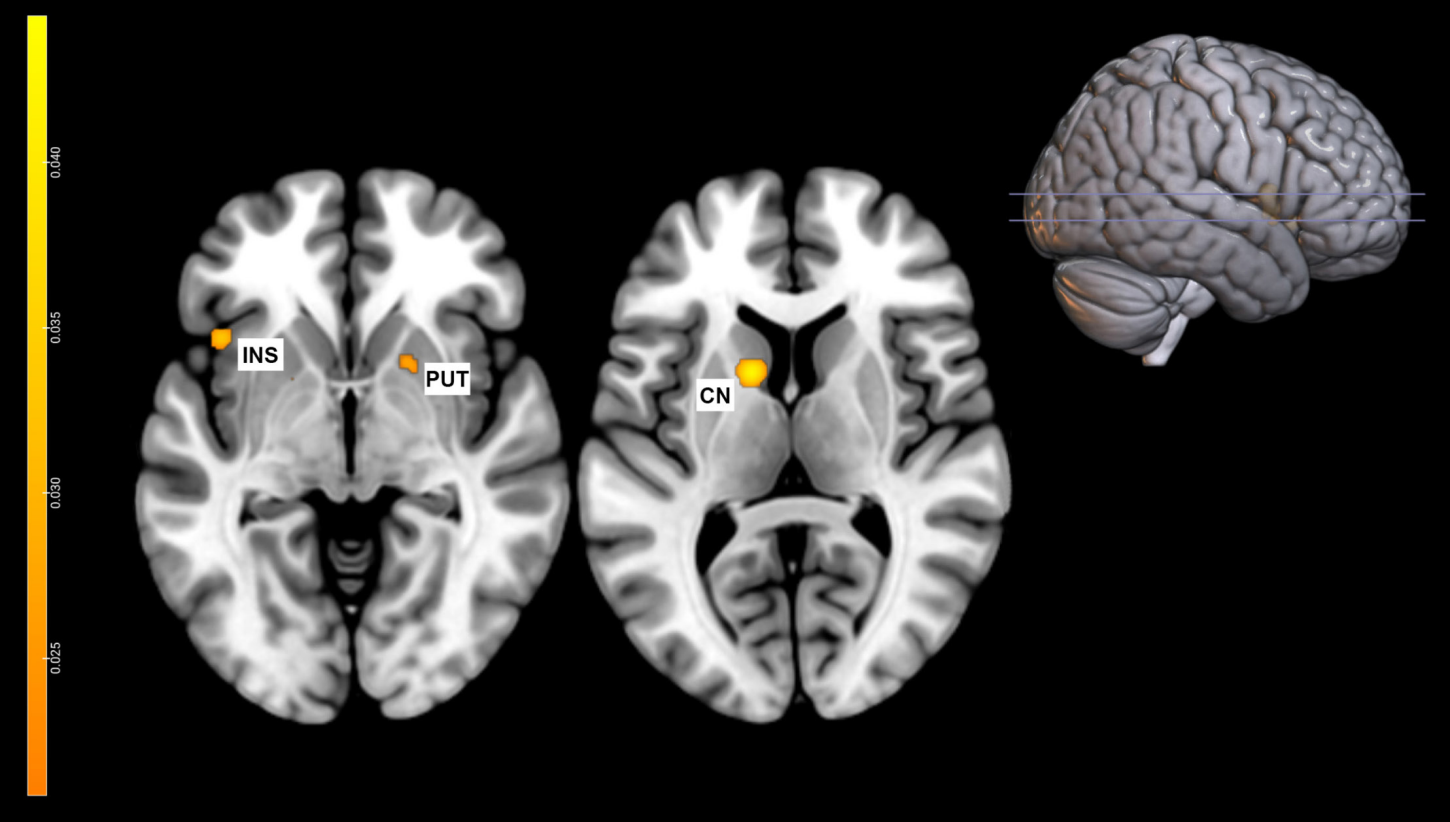
The image shows the results of the MACM analysis of the left caudate. Clusters were found in the left caudate nucleus (CN), left insula (INS), and right putamen (PUT).

The results of MACM conducted on the right IFG and right ACC of the general inhibition, and on the right ACC of the correct motor inhibition did not show any significant results. On the other hand, MACM on the left caudate derived from the correct motor inhibition analysis revealed significant connectivity of this brain region with the left caudate extending up to the left putamen (cluster 1), the left insula (cluster 2) and the right putamen (cluster 3) (Table 3 and Figure 3).

**TABLE 3:** MACM results: cluster of co‐activation associated with the left caudate during correct motor inhibition. From the left, the table reports number, region, hemisphere, volume in mm^3^, ALE score, and coordinates for each cluster.

Cluster #	Region	Hemisphere	Volume (mm^3^)	ALE score	x	y	z
1	Caudate	L	1304	0.044	−12	6	8
1		L		0.032	−18	8	2
2	Insula	L	416	0.033	−42	18	−4
3	Putamen	R	232	0.027	18	10	−4

## 4 Discussion

This study utilized a meta‐analytic approach to clarify the brain circuits that are primarily involved in different types of response inhibition in children and adolescents with ADHD. Four analyses were carried out: one including both types of response inhibition, that is, with and without interference suppression; one analysis focused on interference inhibition, that is, involving conflict (C) tasks; and two analyses focused on motor inhibition, involving no‐conflict (no‐C) tasks, one irrespective of correct/incorrect performance, and one including only correct responses. We also conducted a connectivity analysis using MACM. This approach allowed us to obtain significant convergence of activity across the available studies to disentangle the brain contribution to various response inhibition processes in ADHD.

The first analysis examined general response inhibition, regardless of the inhibition or task type (both C and no‐C), and response accuracy. This analysis identified activation clusters that have previously been associated with response inhibition in individuals with ADHD, namely, the right IFG, the ACC, and the caudate nuclei (e.g., Massat et al. 2018; Zamorano et al. 2017; for reviews, see Hart et al. 2013; Lei et al. 2015). This finding confirms a key contribution played by these regions in general response inhibition, with or without conflict. More importantly, targeted analyses of interference inhibition or motor inhibition (and correct motor inhibition) further dissociated these activation clusters. The right IFG cluster was found to contribute only to interference inhibition, while the other clusters, ACC and caudate, resulted only in the motor/correct motor inhibition analyses. This highlights the specific role played by each region during the different types of inhibition, that is, with or without interference suppression.

The interference inhibition analysis revealed a single activation cluster located in the grey matter, namely, in the right IFG. This suggests that children and adolescents with ADHD commonly activate this area during response inhibition tasks with conflict, regardless of the correctness of the response. While previous studies have suggested the involvement of this brain area during no‐C tasks (e.g., Choo et al. 2022; Breitling‐Ziegler et al. 2021; for a review, see Aron and Poldrack 2005), the present analysis, leveraging the overall available literature, revealed a convergence of activity in the right IFG cluster during C tasks but not during no‐C tasks. This finding is in line with the general role of the IFG in the executive functions (Hampshire et al. 2010), as C tasks are inherently more demanding than no‐C tasks. The involvement of the IFG in response inhibition and, more generally, in cognitive and behavioural control is well‐established (Hart et al. 2013; Lei et al. 2015; Nardo et al. 2011; Santangelo et al. 2009; Santangelo and Macaluso 2013). Furthermore, Zhang et al. (2017) conducted a meta‐analysis on neurotypical individuals and found that this area is active during interference resolution tasks (i.e., C tasks). Other studies have also shown that this region plays a core role in ADHD (e.g., Zamorano et al. 2017; Shulz et al. 2005; Hart et al. 2013). For instance, Zamorano et al. (2017) used the multi‐source interference task (i.e., a C task) to investigate response inhibition in individuals with ADHD. Participants were instructed to press a response button based on the stimuli presented on the screen, either with or without distractors (incongruent and congruent conditions, respectively). The comparison between ADHD vs. TD participants revealed higher activation of the right IFG in ADHD adolescents. The authors suggested that this activation may represent a compensatory mechanism. Individuals with ADHD may require greater cognitive effort to achieve the same level of performance as controls, which may translates into greater metabolic activity in the IFG. This interpretation is in line with the findings of Bos et al. (2017) who observed increased activity in the right IFG in individuals with ADHD as compared to TD during resting state fMRI. Additionally, our finding is also consistent with the evidence that in neurotypical subjects interference/conflict suppression is related to the activation of the fronto‐parietal network, including the IFG (e.g., Grandjean et al. 2012; Siemann et al. 2018). However, although studies on ADHD patients have found a parietal contribution to C tasks (e.g., L.‐Y. Fan et al. 2014; H.‐C. Fan et al. 2017), our analysis did not reveal any posterior clusters. This suggests that the significant common activity in ADHD children and adolescents is more concentrated in the frontal node of this network during the execution of C tasks. The parietal cortex may contribute more to the recruitment of the attentional resources required to carry out this type of task, rather than to the inhibition of response per se (see, e.g., Bianco et al. 2021).

The analyses conducted on no‐C tasks highlighted the contribution of well‐known regions involved with response inhibition (Zhang et al. 2017; Hart et al. 2013). Specifically, the left caudate cluster resulted from both the motor inhibition and correct motor inhibition analyses, indicating that this brain area generally contributes to response inhibition in no‐C tasks in individuals with ADHD, regardless of whether inhibition is correctly acted upon or not. In children and adolescents with ADHD, the left caudate is often activated when they are required to inhibit a response without conflicting information (i.e., no‐C tasks). This is consistent with the evidence that this area matures progressively with growth (Lei et al. 2015; McCarthy et al. 2013). No‐C tasks typically require participants to withhold a motor action, such as pressing a button, when presented with a specific cue. The involvement of the caudate nuclei in this type of task is consistent with their well‐known role in executing movements (Çirak et al. 2021), as well as other cognitive processes, such as response inhibition (Grahn et al. 2008; Alam et al. 2018). These subcortical structures are key regions for response inhibition in both neurotypical subjects (Casey et al. 2001; Zhang et al. 2017) and individuals with ADHD (e.g., Passarotti et al. 2010). For instance, Massat et al. (2018) found greater activation of the basal ganglia during the stop signal task (i.e., a no‐C task) in ADHD children compared to TD, suggesting—again—a compensatory account. This may also explain why we observed the contribution of both caudate nuclei, that is, on the left and right hemispheres, when considering correct response inhibition.

Along with these subcortical structures, we found that the right ACC also played a role in correct response inhibition for no‐C tasks. This medial frontal area is known to be connected with the caudate nucleus (Robinson et al. 2012; Çirak et al. 2021) and involved in emotional functions (for reviews, see Rolls 2019; Etkin et al. 2011), as well as in wide range of cognitive control processes (for a review, see Vassena et al. 2017), including conscious error detection and conflict monitoring (Botvinick et al. 2004; Carter and van Veen 2007; Orr and Hester 2012), behavioural switching (Hikosaka and Isoda 2010), outcome predictions (Alexander and Brown 2011), control resources allocation (Shenhav et al. 2013), and salient stimuli detection (Daviddi et al. 2022; Menon 2015). ACC is a key region of cognitive control that also contributes to inhibitory processes in both neurotypical individuals (Zhang et al. 2017) and those with ADHD (Hart et al. 2013). Furthermore, evidence suggests that the level of activation in this region is proportional to the difficulty in successfully inhibiting a response. Garavan et al. (2002) conducted a combined fMRI‐electroencephalogram study on neurotypical individuals using a classic go‐no go paradigm (i.e., no‐C task). The authors categorized the successful inhibitions as either “difficult” or “easy” based on the speed of correct go responses that immediately preceded the successful withhold action. The results indicated that the response inhibition is generally right‐lateralized. Interestingly, the successful inhibition that was categorized as “difficult” was related to a greater activation of the ACC. The present finding may therefore be linked to specific difficulty in correctly accomplishing the no‐C task in children and adolescents with ADHD. In general, it might be reasonable to suggest that the convergence of the activity in the right ACC during successful inhibition may be due to the additional attentional and monitoring effort needed to correctly withhold actions for individuals with ADHD. In other words, they require more cognitive monitoring to successfully withhold actions.

Finally, a significant MACM result was found in the left caudate (cf. cluster 1 of correct motor inhibition analysis), indicating enhanced functional connectivity with the left insula and right putamen. These areas were found to be functionally connected (Ghaziri et al. 2018) and to be involved in successful response inhibition (for reviews, see Zhang et al. 2017; Hart et al. 2013). The putamen is a subcortical structure that plays a significant role in motor control, as clearly highlighted in several patient studies (e.g., Parkinson's Disease; for reviews, see Meder et al. 2019; Castelnovo et al. 2023). In contrast, the insula is a brain structure that is involved in various systems (for a review, see Pavuluri and May 2015), including cognitive control, with a specific role in detecting salient stimuli (Menon and Uddin 2010). The connection with this structure may therefore emphasize the monitoring of context in search for salient, relevant “stop signals”, which are then communicated to the basal ganglia nuclei (i.e., the putamen and caudate nuclei) to withhold the response.

## 5 Limitations and Conclusions

Some limitations should be noted. The first one is intrinsic to the meta‐analytic approach, as the obtained results are restricted to the studies included in the meta‐analysis. Similarly, it must be considered that the small number of studies included in the MACM analysis is limited to the Sleuth database. Moreover, we did not distinguish between the different subtypes of ADHD because not all the studies included this information or reported the coordinates of activation that differentiated the subtypes. Furthermore, here we characterize the neural correlates of response inhibition by grouping together tasks with and without interference/conflict, while other possible classifications may be adopted in future research to uncover these mechanisms (e.g., by grouping tasks involving motor actions vs. motor cancellation). Additionally, we did not consider the different types of stimuli used in the studies, which included both visual and auditory (verbal) stimuli. This could result in a mix of both perceptual and semantic effects in the observed activations. Furthermore, although most of the papers selected for the current analyses included ADHD patients with a minimum of 18 h of washout, 3 studies (i.e., Beauregard and Lévesque 2006; Cerullo et al. 2009; Zamorano et al. 2017) did not require ADHD participants who were taking pharmacological treatment to undergo a washout period. Instead, they simply asked to discontinue their medication on the day of the task administration. Finally, we were unable to investigate the convergence of activity in children and adolescents with ADHD for correct response inhibition during C tasks due to the insufficient number of contrasts.

Despite these limitations, which could be addressed in future research, here we applied an ALE meta‐analytic approach to elucidate the neural correlates of response inhibition processes in children and adolescents with ADHD. The general inhibition analysis revealed the involvement of fronto‐striatal clusters that overlapped with regions identified in subsequent, and more specific analyses. These regions were then found to play a specific role according to the type of inhibition task, whether it was C or no‐C. In the interference inhibition analysis (i.e., C tasks), the right IFG was identified, while the right ACC and bilateral caudal nuclei were associated with motor inhibition and correct motor inhibition analyses (i.e., no‐C tasks). Ultimately, our findings indicated that frontal‐striatal regions play a central role in response inhibition in ADHD children and adolescents, highlighting a selective role for the IFG in interference and conflict suppression, and the contribution of the ACC and caudate nuclei in no‐conflict tasks, especially during successful motor response inhibition.

## Author Contributions

**Sarah Daviddi:** data curation, formal analysis, investigation, methodology, validation, visualization, writing – original draft, writing – review and editing. **Valerio Santangelo:** conceptualization, methodology, project administration, supervision, visualization, writing – original draft, writing – review and editing.

## Ethics Statement

The authors have nothing to report.

## Conflicts of Interest

The authors declare no conflicts of interest.

## Peer Review

The peer review history for this article is available at https://www.webofscience.com/api/gateway/wos/peer‐review/10.1111/ejn.70244.

## Data Availability

The data that support the findings of this study are available from the corresponding author upon reasonable request.
